# Supplementary material for: BCG Vaccination-Associated Lower HbA1c and Increased CD25 Expression on CD8+ T Cells in Patients with Type 1 Diabetes in Ghana
Source: Vaccines (Basel). 2024 Apr 24;12(5):452. doi: 10.3390/vaccines12050452 (PMC11125916; doi:10.3390/vaccines12050452)
Supplement: Supplementary file 1 [file vaccines-12-00452-s001.zip › vaccines-2953637-supplementary.pptx]

## Slide 1
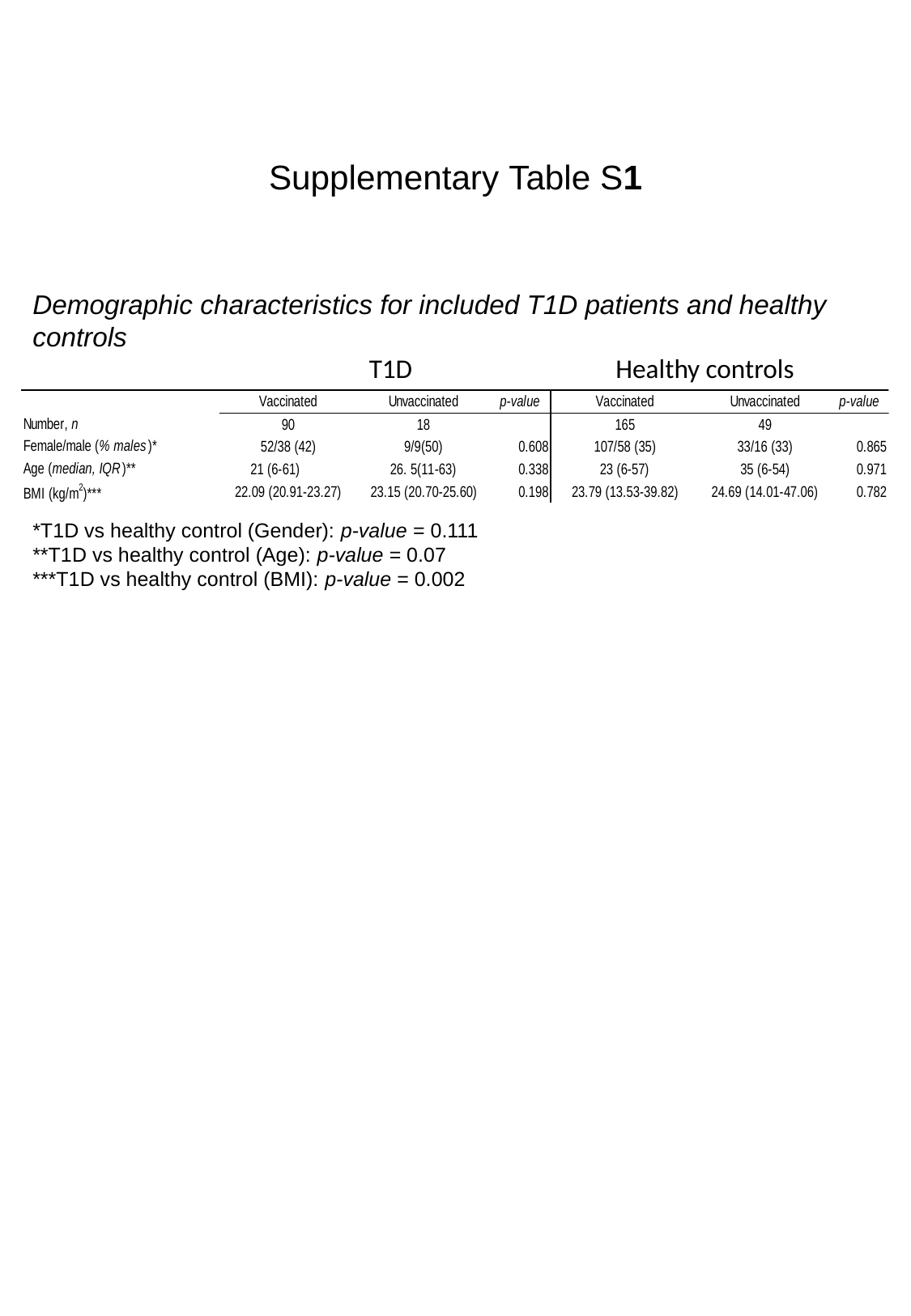

Supplementary Table S1
Demographic characteristics for included T1D patients and healthy controls
T1D
Healthy controls
*T1D vs healthy control (Gender): p-value = 0.111
**T1D vs healthy control (Age): p-value = 0.07
***T1D vs healthy control (BMI): p-value = 0.002

## Slide 2
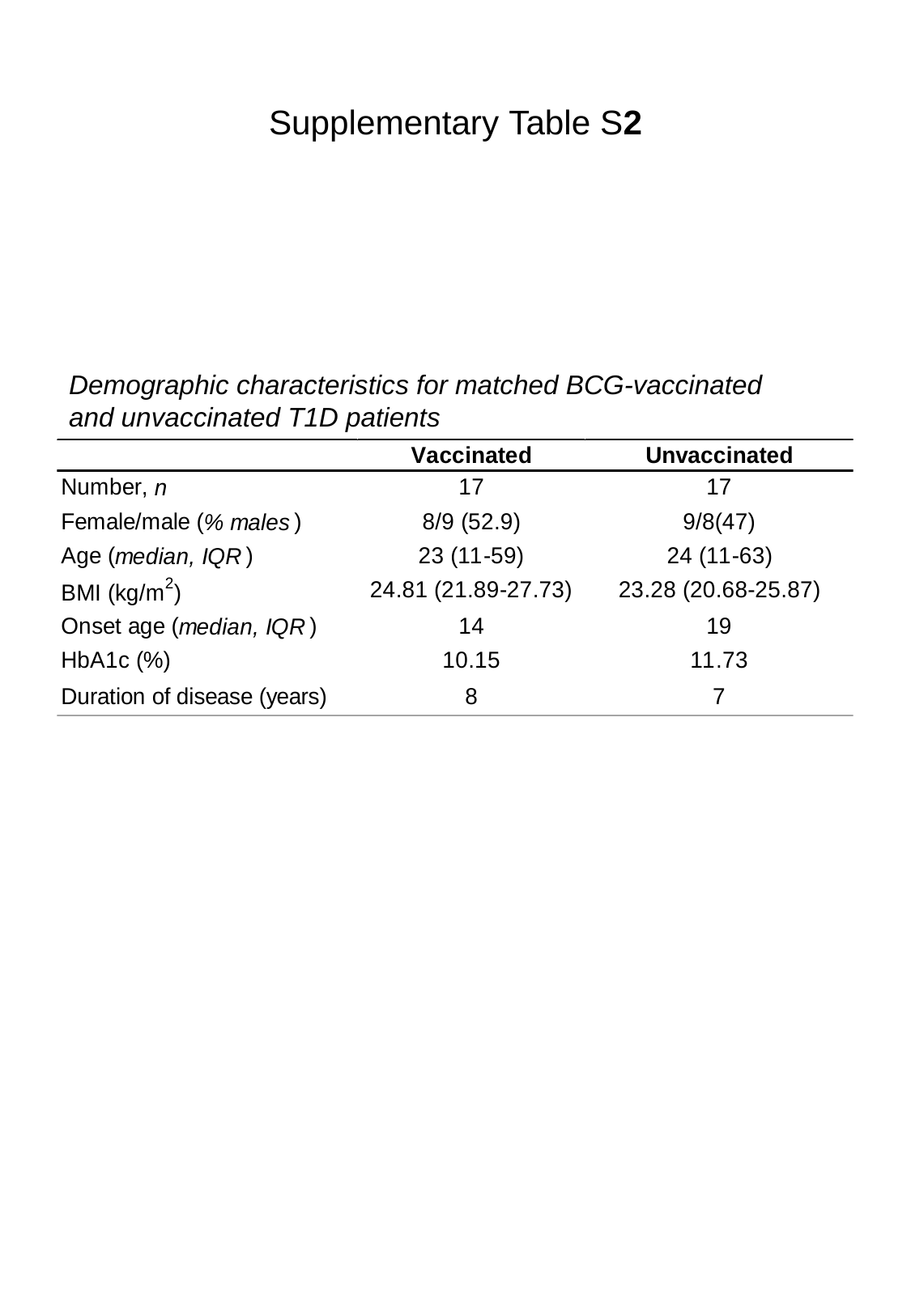

Supplementary Table S2
Demographic characteristics for matched BCG-vaccinated and unvaccinated T1D patients

## Slide 3
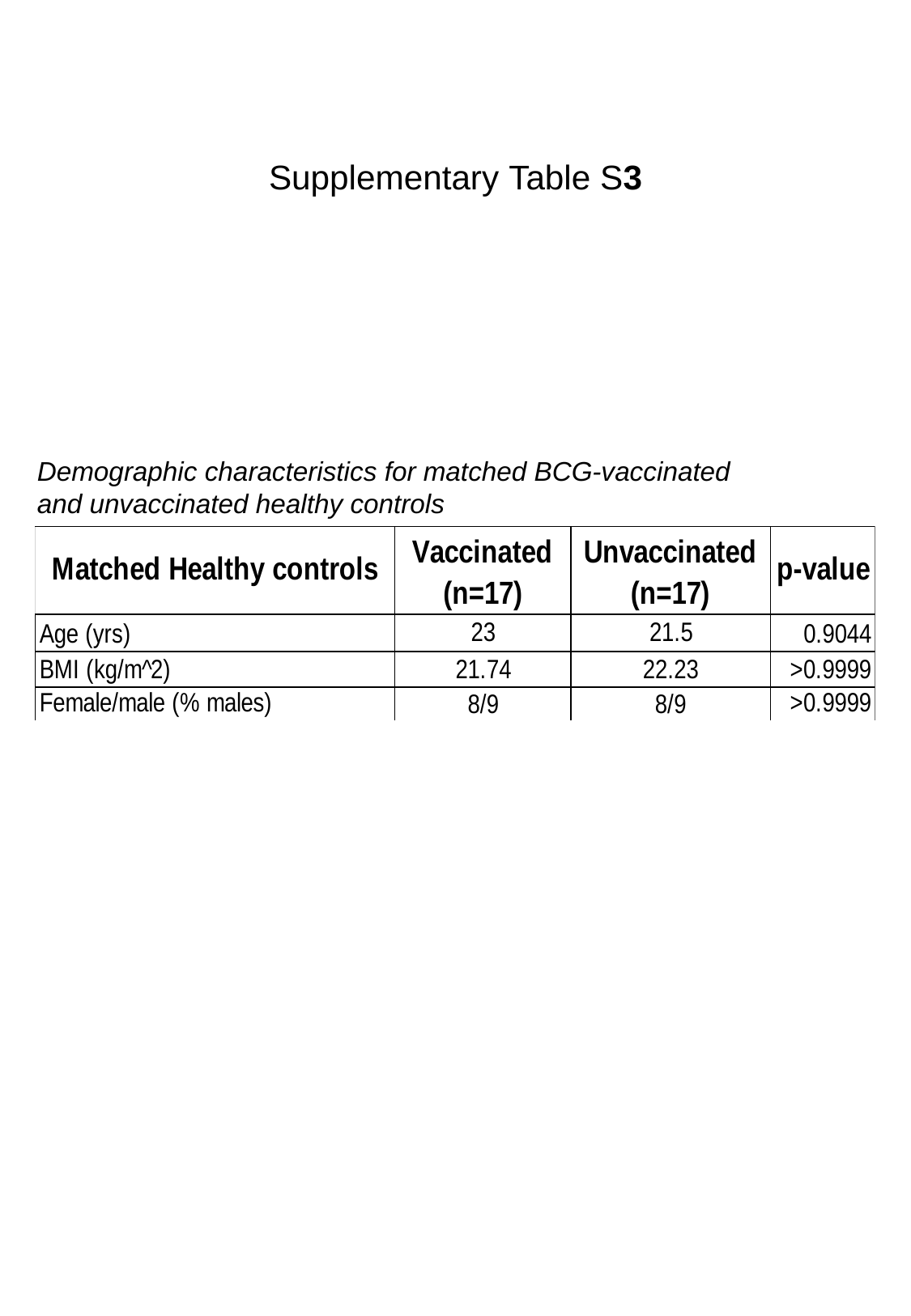

Supplementary Table S3
Demographic characteristics for matched BCG-vaccinated and unvaccinated healthy controls

## Slide 4
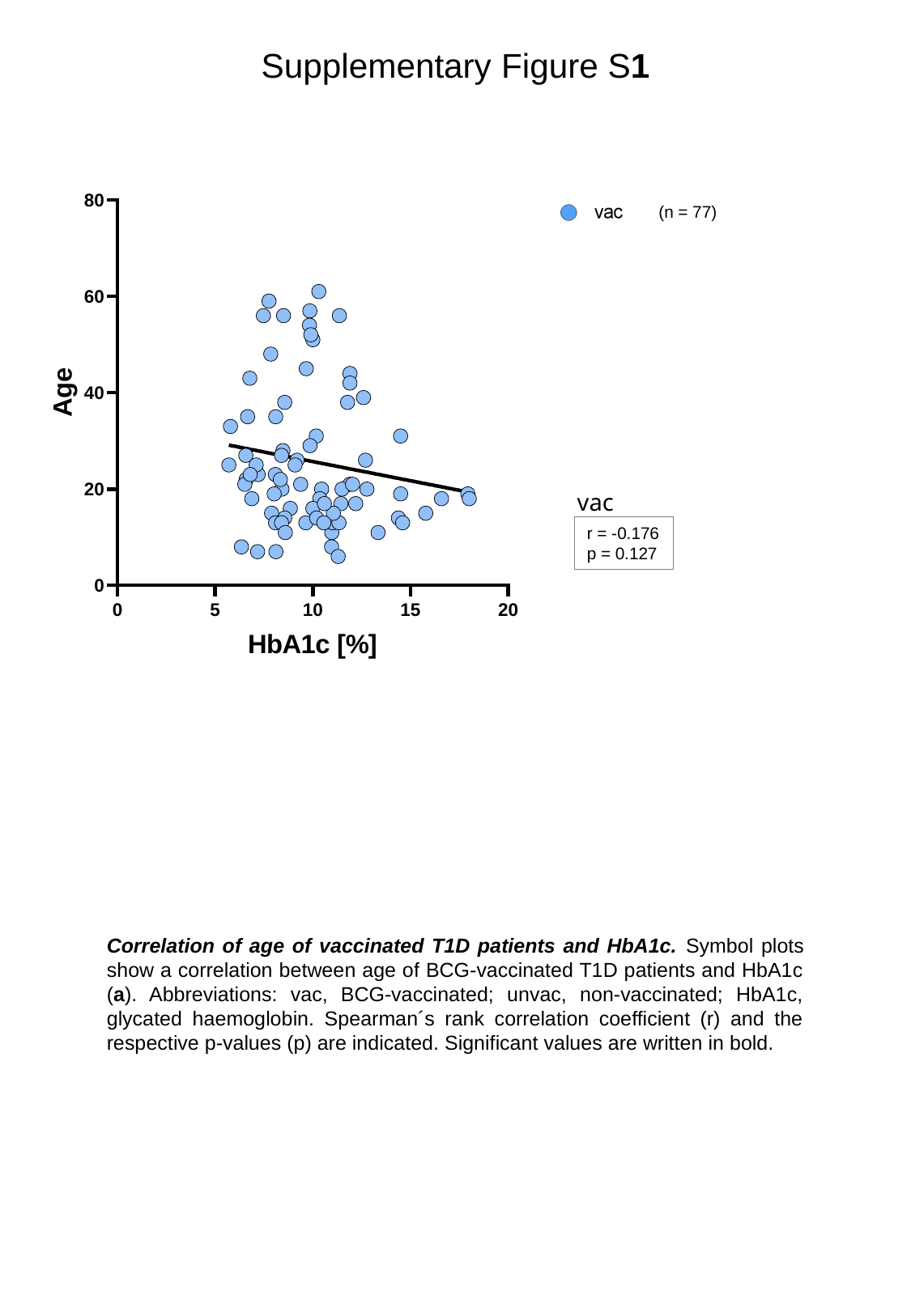

Supplementary Figure S1
(n = 77)
vac
r = -0.176
p = 0.127
Correlation of age of vaccinated T1D patients and HbA1c. Symbol plots show a correlation between age of BCG-vaccinated T1D patients and HbA1c (a). Abbreviations: vac, BCG-vaccinated; unvac, non-vaccinated; HbA1c, glycated haemoglobin. Spearman´s rank correlation coefficient (r) and the respective p-values (p) are indicated. Significant values are written in bold.

## Slide 5
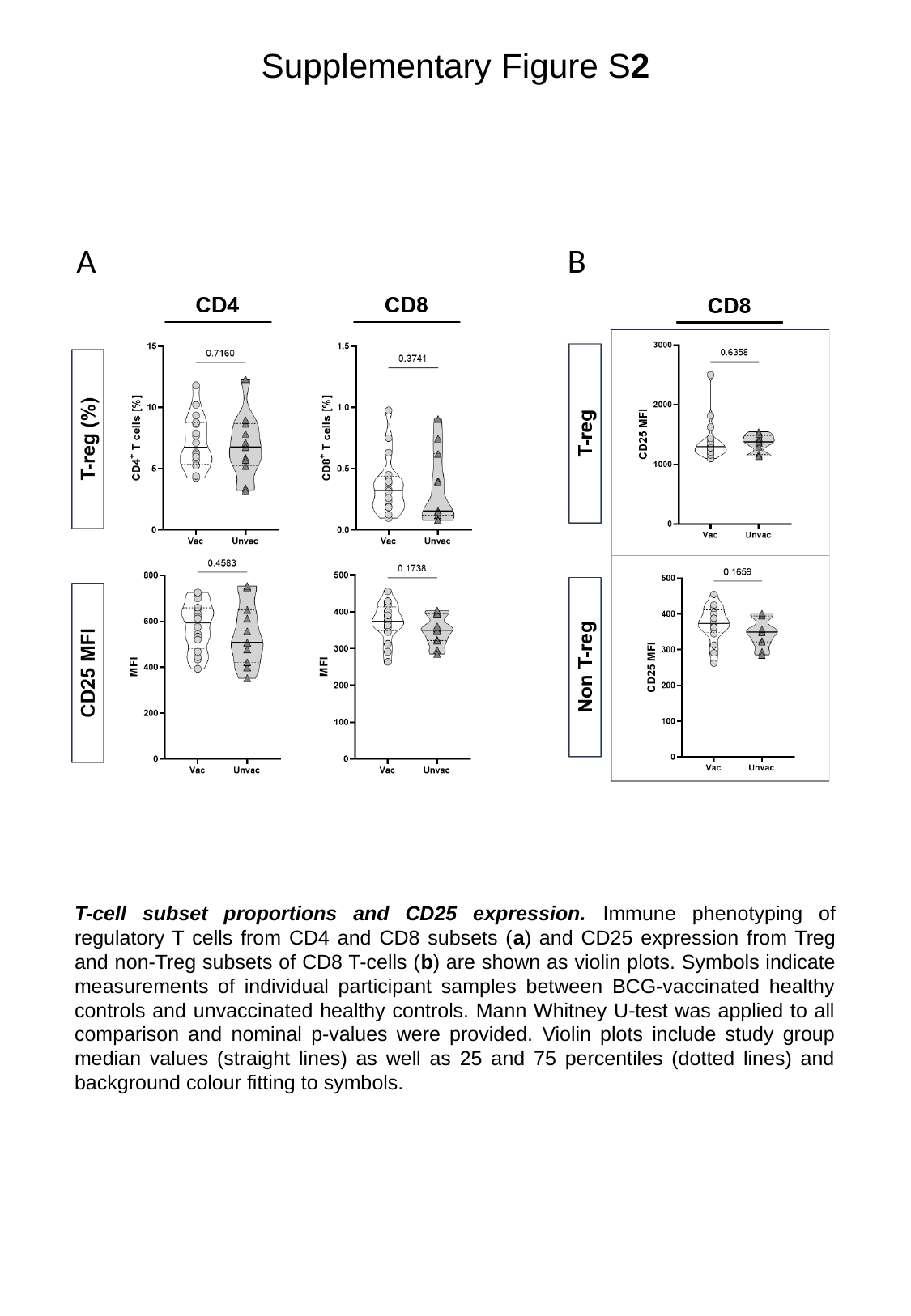

Supplementary Figure S2
A
B
T-cell subset proportions and CD25 expression. Immune phenotyping of regulatory T cells from CD4 and CD8 subsets (a) and CD25 expression from Treg and non-Treg subsets of CD8 T-cells (b) are shown as violin plots. Symbols indicate measurements of individual participant samples between BCG-vaccinated healthy controls and unvaccinated healthy controls. Mann Whitney U-test was applied to all comparison and nominal p-values were provided. Violin plots include study group median values (straight lines) as well as 25 and 75 percentiles (dotted lines) and background colour fitting to symbols.

## Slide 6
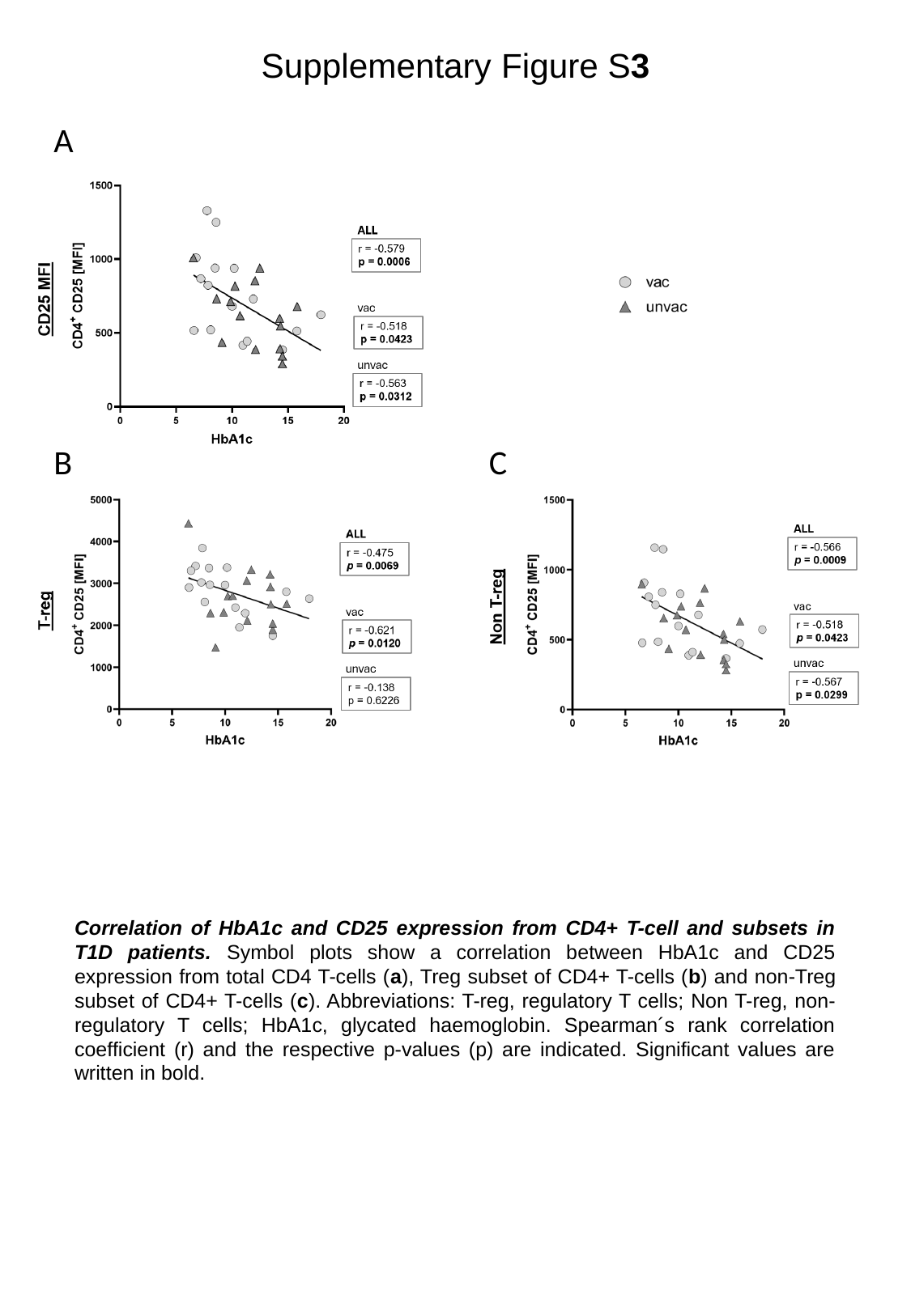

Supplementary Figure S3
A
C
B
Correlation of HbA1c and CD25 expression from CD4+ T-cell and subsets in T1D patients. Symbol plots show a correlation between HbA1c and CD25 expression from total CD4 T-cells (a), Treg subset of CD4+ T-cells (b) and non-Treg subset of CD4+ T-cells (c). Abbreviations: T-reg, regulatory T cells; Non T-reg, non-regulatory T cells; HbA1c, glycated haemoglobin. Spearman´s rank correlation coefficient (r) and the respective p-values (p) are indicated. Significant values are written in bold.
